# Ivy’s Applied Anatomy and Oral Surgery

**Published:** 1913-06-15

**Authors:** 

**Affiliations:** Philadelphia


### BIBLIOGRAPHY.

### IVY'S APPLIED ANATOMY AND ORAL SURGERY.

Dr. Ivy's book is just exactly what the practicing dentist has long wanted—a concise work on applied anatomy and oral surgery—written with their sole needs in mind. We do not know of any man better fitted to write such a book than Dr. Ivy, who is a graduate in both dentistry and medicine. The illustrations add greatly to the text, which is itself unusually graphic. Price $1.50

W. B. SAUNDERS COMPANY, Philadelphia.

